# Viscoelasticity as a measurement of clot structure in poorly controlled type 2 diabetes patients: towards a precision and personalized medicine approach

**DOI:** 10.18632/oncotarget.10618

**Published:** 2016-07-15

**Authors:** Etheresia Pretorius, Janette Bester

**Affiliations:** ^1^ Department of Physiology, University of Pretoria, Pretoria, South Africa

**Keywords:** type 2 diabetes, viscoelasticity, coagulation, precision medicine, personalized medicine approach, Pathology Section

## Abstract

**Objectives:**

Type 2 diabetes patients (T2D) have a considerably higher cardiovascularrisk, which is closely associated with systemic inflammation, and an accompanying pathologic coagulation system. Due to the complexity of the diabetic profile, we suggest that we need to look at each patient individually and particularly at his or her clotting profile; as the healthiness of the coagulation system gives us an indication of the success of clinical intervention.

**Results:**

T2D coagulability varied markedly, although there were no clear difference in medication use and the standards of HbA1c levels.

**Research design and methods:**

Our sample consisted of 90 poorly controlled T2D and 71 healthy individuals. We investigated the medication use and standards of HbA1c levels of T2D and we used thromboelastography (TEG) and scanning electron microscopy (SEM) to study their clot formation.

**Conclusion:**

The latest NIH guidelines suggest that clinical medicine should focus on precision medicine, and the current broad understanding is that precision medicine may in future, provide personalized targets for preventative and therapeutic interventions. Here we suggest a practical example where TEG can be used as an easily accessible point-of-care tool to establish a comprehensive clotting profile analysis for T2D patients; and additionally may provide valuable information that may be used in the envisaged precision medicine approach. Only by closely following each individual patient's progress and healthiness and thereby managing systemic inflammation, will we be able to reduce this pandemic.

## INTRODUCTION

The world-wide prevalence of cases of type 2 diabetes and pre-diabetes have been spiralling out of control, and not only in developed countries, but also in third-world countries [1–4], with more than half a billion cases expected by 2030 [5]. Co-morbidities are also a major health burden on all health systems, and include hypoglycaemia, hypertension, dyslipidaemia, cardiovascular and heart related deaths, stroke, kidney disease, eye problems and also amputations [6]. Cardiovascular risk, vascular dysfunction, accelerated atherosclerosis and changed inflammatory marker profiles are closely associated with systemic inflammation; and this is also the case in type 2 diabetes individuals [7–16]. A chronic state of systemic inflammation also appears to be a central mechanism underlying the pathophysiology of insulin resistance and metabolic syndrome [16] and consequently also in type 2 diabetes. Most type 2 diabetes patients are on a plethora of medication including medication for comorbidities, and will need incrementally more complex therapeutic regimens to control hyperglycaemia as the disease progresses [17]. Cardiovascular disease and a pathological coagulation system, therefore remains the leading cause of mortality in patients with this condition [15].

Previously we showed that pathophysiological erythrocytes (RBCs), platelets and atypical fibrin fibre formation seen as an altered fibrin structure, are important hallmarks of inflammation [18–22], and these changes are also found in type 2 diabetes [7, 23, 24]. Diabetic fibrin fibres have a netted and matted appearance, and previous research suggested that in the condition, either hypercoagulability [25–27], reduced clot permeability and decreased susceptibility of clot to (fibrino)lysis may happen [21, 28–31]. Also, in type 2 diabetes, increased glucose levels may play an important role in fibrin fibre packaging, where glycation of fibrinogen may be a key culprit in a changed fibrin structure [32–34].

In this paper we looked at thromboelastic parameters using thromboelastography (TEG) and scanning electron microscopy (SEM) of an arbitrarily chosen poorly controlled patient sample, to see if the results could be used to determine if there is variability in the coagulation statuses of these patients. A typical generalized clinical treatment approach is usually to determine the level of hyperglycaemia and the presence of comorbidities (e.g. hyperlipidaemia and hypertension), followed by treatment of each identified complication. However, the coagulation profile is indicative of inflammatory status of an individual, and by tracking the coagulatory profile closely, the patient's medication may be streamlined. This will allow the clinician to also determine the general inflammatory healthiness, ultimately resulting in a better-controlled condition. Here we argue that we are currently not following the right treatment approach, and in many cases, we fail in tracking the effects of the medication in the condition. To successfully implement and track a treatment regime that not only improves glucose regulation, but also reduces systemic inflammation, we need to follow an individualised approach. Information from viscoelastic parameters may play an fundamental role in such a precision-medicine approach, together with genome-based and other molecular techniques, as suggested by the NIH [35]. To address the increasing diabetes prevalence, we need to investigate the use of novel technologies; and in this approach, individualized disease tracking, forms an essential part in the diagnosis, as well as treatment.

## RESULTS

Tables 1, 2, 3 show comprehensive sample data. We included all individual data of the 2 groups, to make the point that the individual type 2 diabetes sample results are greatly variable. From our diabetes sample of 90, 46 were randomly chosen for TEG analysis. A Tukey analysis was performed (it compares the means of every TEG parameter to the means of every other parameter) and all comparative p-values were P > 0.9999 (this will be discussed in detail later).

**Table 1:** Demographics and medication usage and TEG of PPP of healthy individuals (*n*=71)

No.	G	Age	R	K	Angle	MA	MRTG	TMRTG	TTG
1	M	22	9	9.7	53.1	20.7	1.71	10.7	131.1
2	M	61	8.2	6.4	49.6	21.8	2.58	11.2	139.9
3	M	20	9.6	6.1	49.1	26	1.53	12.1	142.5
4	M	33	7.8	3.4	55.7	54.6	4.56	13.5	602.5
5	M	18	11.8	6.7	52.3	25.1	1.71	15.4	167.8
6	M	17	7.7	2.5	71.9	24.3	5.6	8.6	160.7
7	M	47	2.5	2.1	73.1	32.6	17.76	5.1	240.1
8	M	32	11.1	8.1	46.8	21.9	2.79	7.3	219.4
9	M	20	7.9	7.2	45.8	26	2.06	11.1	175.7
10	M	30	11.2	4.8	49.5	30.5	2.64	14.3	219.3
11	M	31	10.8	6.3	48.6	28	1.75	14.3	194.3
12	M	47	7.6	2.8	60.6	32.8	5.74	10.3	244.5
13	M	53	11.2	6.1	49.5	29.2	2.33	14.6	206.3
14	M	24	11.2	6.8	47.5	25	2.36	13.6	166.8
15	M	23	9.3	14.2	44.6	20.3	1.31	12.8	128.0
16	M	23	10.8	1.7	71.2	35.8	8.9	12.4	279.1
17	M	23	14.5	4.4	47.3	29.6	3.4	18.3	210.6
18	M	22	12.1	6.6	52.5	21.3	2.84	13.1	135.7
19	M	23	9.3	6	66.5	21.2	7.08	10.3	134.5
20	M	23	10.8	7.6	65.7	20.9	2.6	12.4	132.2
21	M	23	7.7	4.6	72.9	21	5.39	8.5	133.4
22	M	23	7.8	2.9	66.5	27.7	4.55	8.9	192.2
23	M	30	6.7	4.2	73	22.1	5.59	7.4	142.0
24	M	23	6.6	2.3	68.7	26.6	4.55	7.6	181.4
25	M	23	14	9.2	50.1	28	1.43	15.3	194.9
26	M	28	8.9	3.6	59.5	35.4	6.45	13.5	273.9
27	M	20	10.2	11.4	50.3	22.2	1.53	12.1	142.5
28	M	20	4	2.9	60.2	37	4.45	5.8	294.3
29	M	19	7.1	1.4	74.6	35.5	12.24	8.7	276.8
30	M	19	7.9	3.5	61.1	35.6	6.43	12.0	278.4
31	M	19	11.9	11.2	59.9	20	2.61	13.2	125.2
32	M	20	9.5	4.4	61.5	22.6	3.56	10.9	146.0
33	M	52	6.8	1.7	71.8	39.9	10.37	8.7	333.0
34	M	77	5.5	1.3	76.5	32.6	7.77	6.3	243.3
35	M	84	18.8	3.6	54.4	31.9	3.59	21.4	234.3
36	M	88	6.6	2.2	67.6	28.3	5.67	8.1	198.0
37	F	28	9.9	8.1	50.8	23.5	2.01	8.9	143.5
38	F	18	3.4	1.3	76	31.4	7.55	4.4	241.8
39	F	24	7.3	3.6	67.3	37.9	3.89	8.3	305.4
40	F	19	4.2	3	69.3	27	5.13	5.3	185.7
41	F	66	4.1	1.5	74.7	32.9	7.5	5.0	247.2
42	F	48	6.2	3.1	61.6	35.2	20.07	8.6	471.6
43	F	29	6.6	3	59	30.9	4.46	8.9	222.3
44	F	48	8.2	4.6	61.3	28.2	2.66	9.3	195.5
45	F	32	3.2	2.7	62.5	28.6	5.83	5.2	201.6
46	F	46	9.8	2.1	67.4	32.3	5.94	11.6	238.9
47	F	24	4.9	1.2	77.6	31.5	8.85	5.8	253.5
48	F	48	7.8	4.3	52.3	26.2	3.69	10.4	179.3
49	F	18	9.6	15.8	43.4	20	1.36	12.4	127.1
50	F	55	4.1	1.5	74.7	32.9	7.5	5.0	247.2
51	F	23	12.1	3.6	56.3	24.7	3.83	14.2	164.2
52	F	24	8.3	1.8	71.6	34	6.25	9.4	249.4
53	F	30	11.5	7.4	51.9	25.9	1.82	13.6	175.4
54	F	29	6.8	5.8	60.1	28.6	3.58	8.2	207.1
55	F	20	7.3	8.3	67.7	20.4	4.19	8.3	128.6
56	F	28	9.8	2.1	69	27.3	5.85	11.0	188.0
57	F	19	9.7	5.1	61.1	21.7	3.33	11.1	138.9
58	F	31	10.2		58	19.5	2.84	11.8	121.8
59	F	40	5.5	1.6	71.9	34.1	7.91	6.8	259.7
60	F	43	6.8	3	64.7	46.2	13.18	11.1	431.8
61	F	46	7.2	1.3	74.3	32.3	8.42	8.5	240.2
62	F	60	8.1	1.7	71.8	37.4	9.63	9.8	299.5
63	F	61	9.8	2.7	61.8	40.1	4.66	12.0	336.1
64	F	61	5.8	3.4	71	27.2	4.41	6.5	187.3
65	F	76	9.2	2.5	66.1	27.5	4.63	10.7	190.7
66	F	86	3.1		33.6	2.4	0.98	2.9	13.1
67	F	87	7.2	2.4	64.1	36.4	5.27	9.5	287.2
68	F	88	8	2	68.9	35	5.44	9.8	269.9
69	F	88	5.6	2	69	31.9	6.31	7.0	234.6
70	F	91	7.8	2.4	65.3	35.2	4.83	9.4	273.1
71	F	92	16.1	3.7	53.7	34.8	4.09	19.7	267.9
**Median**		**29**	**8**	**3.5**	**61.6**	**28.3**	**4.55**	**10.3**	**201.6**
**SD**		**23**	**3.02**	**3.11**	**10.01**	**7.41**	**3.52**	**3.5**	**86.7**

**Table 2:** Demographics and medication usage and TEG of PPP of type 2 diabetes patients

DEMOGRAPHICS			INTERNATIONAL CONVERSION OF NGSP (HbA1c), IFCC AND eAG			MEDICATION USE										
No.	G	Age	NGSP (HbA1c)	OFCC	eAG	CHOL	GLUC	HT	AC							
1	M	68				x	x	x	x							
2	M	56	11.9	107	295		x	x								
3	M	71	8.3	67	192		x	x								
4	M	80	7	53	154	x	x	x	x							
5	M	37	7.6	60	171		x									
6	M	56	7.7	61	174		x	x								
7	F	71	8	64	183	x	x	x	x							
8	F	48	6.2	44	131	x	x	x	x							
9	F	70	7.5	58	169		x	x								
10	F	82	8.9	74	209	x	x	x	x							
11	M	62	5.9	41	123		x	x								
12	M	71	10.5	91	255	x	x	x	x							
13	M	70	10.4	90	252		x	x								
14	F	58	6	42	126		x									
15	F	61	8.2	66	189		x	x								
16	M	56	8.6	70	200	x	x	x	x							
17	M	42	13.6	125	344		x									
18	F	62	10.6	92	258	x	x	x	x							
19	M	41	5.5	37	111	x	x		x							
20	M	63	10.2	88	246	x	x	x	x							
21	F	62	8.2	66	189	x		x								
22	M	52					x	x								
23	F	59	11.6	103	286	x		x								
24	M	73				x	x	x	x							
25	F	62	8	64	183		x	x								
26	F	55	6.3	54	134	x	x	x	x							
27	F	49	13.5	124	341		x	x								
28	F	42	8.3	67	192	x	x	x								
29	F	65	7.3	56	163	x	x	x	x							
30	M	63				x	x									
31	M	51					x									
32	M	57				x	x		x							
33	F	44	6.2	44	131	x		x	x							
34	F	53					x									
35	F	47					x									
36	F	42					x	x	x							
37	F	52	9.5	80	226		x		x							
38	F	60					x	x								
39	F	52	12.7	115	318		x									
40	F	50					x									
41	F	52														
42	M	60					x									
43	F	56	5.7	39	117	x	x	x	x							
44	M	54	7.7	61	174		x	x	x							
			INTERNATIONAL CONVERSION OF NGSP (HbA1c), IFCC AND eAG			MEDICATION USE				TEG PARAMETERS FOR PPP						
No	G	Age	NGSP (HbA1c)	IFCC	eAG	CHOL	GLUC	HT	AC	R	K	ANGLE	MA	MRTG	TMRTG	TTG
45	F	61								9.6	4.8	47.7	56.4	3.8	17.3	647.5
46	F	59	6.8	51	148		x			12.2	4.4	44.4	53.2	7.5	18.1	569.4
47	F	59	5.8	40	120	x	x	x	x	11	3.8	60.2	23.5	3.62	12.67	154
48	F	56	11.1	98	272	x	x	x		14.8	2.9	59.4	31.3	4.6	17	226.4
49	F	64	9	75	212		x	x	X	7	0.9	79.1	36.1	14	8.1	282.2
50	F	61	8	64	183	x	x	x	x	23.7	6.8	43.4	35.7	2.1	29.8	278.4
51	F	58	6	42	126	x	x	x	x	13.2	1.5	74.2	46.3	12.8	15	432.1
52	M	60	7.5	58	169	x	x	x	x	8.6	1.8	70.6	36.4	8.4	11.3	869.2
53	F	57	8	64	183	x	x	x	x	16.8	4.1	55.9	51.9	8.7	23.2	539.8
54	F	63	7.6	60	171	x	x	x		10.4	4.5	55.2	53.8	7.7	18.3	584.8
55	M	73	8	64	183	x	x	x		12.8	5.1	40.2	42	6.3	19.8	362.9
56	M	62	9.2	77	217	x	x	x		11.5	17.9	36.8	42.9	0.65	17.33	378.25
57	F	62	11.3	100	278	x	x	x	x	13.9	1.8	71.3	58.5	10.8	17.3	706.3
58	M	49	10.3	89	249		x	x		12.3	1.7	71	45.4	9.2	14	418
59	F	45	11.6	103	286	x	x	x	x	13.2	2.5	63.3	43.6	6.11	16.33	388.3
60	F	49	6.8	51	148	x	x	x		10.1	3.6	54.3	41.8	3.7	13.8	360.8
61	M	72	6.7	50	146		x	x	x	3.17	>20	32.7	2.6	0.18	35.4	13.6
62	M	46	11.6	103	286		x			16.7	1.8	70	55.2	7.44	18.3	623.01
63	M	40	8.7	72	203			x		7.6	3.2	64.4	26.9	3.5	8.8	184.8
64	M	55	5.8	40	120		x	x		38.5	12.8	37	29.5	1.09	43.75	210.6
65	M	80	6.8	51	148	x	x	x		22.1	5.9	44.8	28	2.2	25.9	195.3
66	M	64	?				x			7.6	1.4	74.8	55.1	12.74	9.25	617.9
67	M	72	6.7	50	146		x	x	x	6.8	1.9	69.2	46.8	6	8.4	440.5
68	M	59	?			x	x			5.5	5.1	38.3	47	5.4	11.6	445.5
69	M	75	6.6	49	143		x	x	x	8.5	1.5	74.5	52.2	12.45	10.6	548.6
70	M	41	8.3	67	192	x	x		x	8.6	1.6	72.3	38	9.9	10.3	306.4
71	F	81	9	75	212	x		x	x	34.6	6.2	47	58.6	3.8	42.4	713.5
72	F	53	7.2	55	160	x	x	x	x	47.5*						
73	M	41	11.6	103	286	x	x	x	x	42.9*						
74	F	53	10.9	96	266	x	x	x	X	42*						
75	M	56	12.1	109	301	x	x	x		56.9*						
76	F	70	11.6	103	286	x		x	x	93.4*						
77	M	66	11.9	107	295	x	x	x	x	40.6*						
78	F	54	15.1	142	387	x	x	x		47.4*						
79	M	65	7	53	154	x	x	x		42.6*						
80	F	59								93.1*						
81	M	52	8.1	65	186	x	x	x	x	48.4*						
82	M	52	12.2	110	303	x	x	x		42.7*						
83	F	62	10.2	88	246	x	x	x		52.2*						
84	M	59	8.5	69	197	x	x	x	x	35.8						
85	F	58	10.6	92	258	x	x	x		29.2*						
86	F	66	10	86	240	x	x	x		52*						
87	M	60	12.2	110	303	x	x	x		30*						
88	F	69	7.4	57	166	x	x	x		74.9*						
89	M	58	11.9	107	295	x	x	x	x	28.2*						
90	M	61	6.9	52	151	x	x	x	x	29.5						

From patient 45 we did TEG analysis. Blue bars show *hypocoagulable individuals*; purple bars show *hypercoagulable individuals*; pink bars show *pseudohypocoagulable profile* and tan bar shows *pseudo-standard clotting* profile.

G: Gender

NGSP: HbA1c (%): (< 7%) percentages in red are too high; IFCC: HbA1c (mmol.mol) and eAG (mg.dL-1)

CHOLESTROL MEDICATION (CHOL): Dyslipidaemia: Simvastatin, Lipitor

GLUCOSE CONTROL (GLUC): Metformin / Oral

HYPERTENSION MEDICATION (HT): Coversyl, Amlodopin, Carvedilol, Adalat

ANTICOAGULANT MEDICATION (AC): Aspirin or Disprin

**Table 3:** Median and SD analysis of ALL diabetes patients

	DEMOGRAPHICS	International conversions of NGSP, IFCC and eAG			TEG PARAMETERS FROM PPP						
	Age	NGSP (HbA1c)	IFCC	eAG	R	K	Angle	MA	MRTG	TMRTG	TTG
Median	59.0	8.3	67	192	12.2	3.4	59.4	43.6	6.1	17.0	418.0
SD	± 9.9	± 2.3	± 24.9	± 65.6	± 9.3	± 3.8	± 14.4	± 12.9	± 3.9	± 9.6	± 202.6

TEG parameters were calculated from patient 45, and patient 72 to 90 only have R-values.

**Figure 1:**
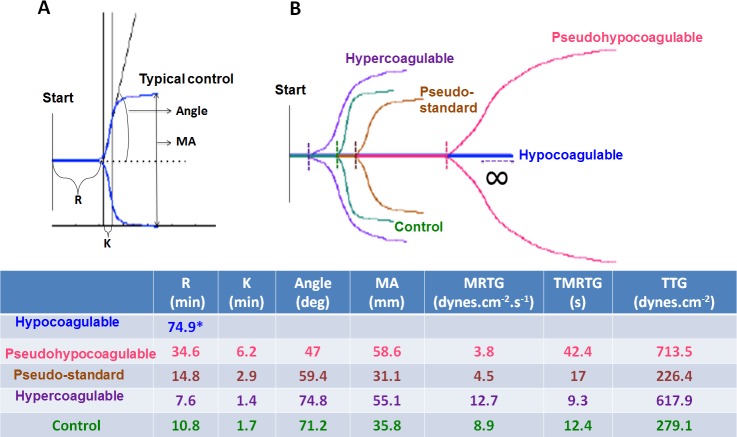
**A.** Healthy control plasma coagulation TEG trace showing the different parameters: R: Reaction time, first measurable clot formation; K: Achievement of clot firmness; Angle: Kinetics of clot development; MA: Maximum clot strength; MRTG: Maximum rate of thrombus generation; TMRTG: Time to maximum rate of thrombus generation; TTG: Final clot strength. **B.** Healthy TEG trace shown in green projected onto the 4 different trace types seen in type 2 diabetes.

**Figure 2:**
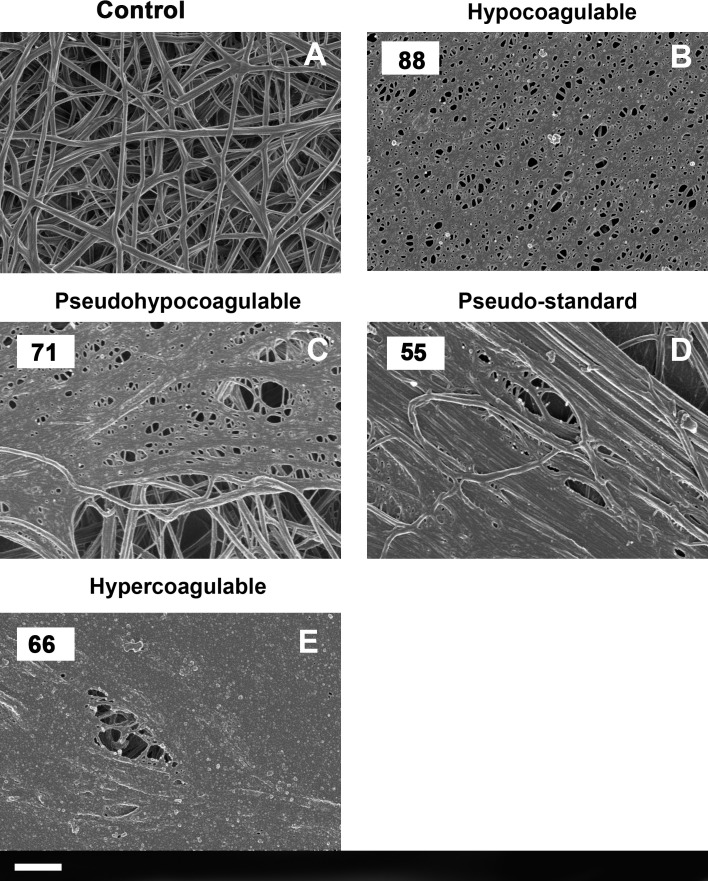
**A.** A typical healthy fibrin fibre net versus **B.** to **E.**) typical type 2 diabetes fibrin fiber nets according to the four types identified with TEG. (Numbers in the top left corner correspond with patient number in Table 3). Scale bar: 1 μm.

TEG of healthy individuals show trends typically associated with healthy viscoelastic parameters [36–38]. SEM micrographs of healthy individuals show well-structured elongated fibres, with open spaces that form a stable clot structure (Figure 2A). This is in line with previous structural analysis of healthy fibrin fibres [7, 19, 39–41]. The diabetes TEG analyses (Figure 1A and 1B) and SEM results Figure 2B to 2E) show four possible trends. These trends are:

1. No R-time established after 30 minutes; no TTG reached (Figure 1B) possible *(hypocoagulable profile).* Clot ultrastructure has a fine-netted appearance, with no visible elongated fibres (Figure 2B). See patient 72 to 90 and discussion in the next paragraphs. This suggests that the PPP never forms a stabilized fibrin clot in the TEG.
2. R-time > 30 minutes, overall increased clot strength, and time to maximum thrombus generation is prolonged, with variable TTG (Figure 1B) *(pseudo-hypocoagulable profile.* SEM showed mainly a fine and netted clot, with limited thick fibres (as typically present in the controls) (Figure 2C). This suggests that the PPP forms a flimsy fibrin clot.
3. R-time from 8 up to 14.9 minutes and TTG or clot strength from 100 to 500 dynes.cm^−2^ (Figure 1B) *(pseudo-standard clotting profile).* SEM showed densely packed elongated fibres, arranged in plates with no fine nets (Figure 2D).
4. R-time < 8 minutes with TTG > 500 dynes.cm^−2^. TMRTG is increased, suggesting an increased clot strength (Figure 1B) *(hypercoagulable profile).* SEM showed matted plates, with limited areas that actually showed individual fibrin fibres or even fine nets (Figure 2E).

## DISCUSSION

Thromboelastography is a technique that shows coagulation kinetics data, and the technique has been extensively utilized in the monitoring of haemostasis during surgery. It is therefore known as a point-of-care instrument, and it can show hypercoagulable and hypofibrinolytic states. We have previously shown that it can be used to study viscoelastic properties in Alzheimer's disease and that these properties correlate with ultrastructural (SEM) analysis of fibrin fibres [42, 43].

Type 2 diabetes is classified as an inflammatory condition, and most patients have glucose dysregulation and cardiovascular comorbidities, including hyperlipidaemia and hypertension. Inflammatory conditions are known to affect clotting, fibrin structure and also erythrocyte function and structure [18–22, 44]. In type 2 diabetes, this hypercoagulability is also mediated by enhanced erythrocyte pathology, which in turn, could promote thrombosis and hypercoagulation [7, 23, 24, 45]. Platelets are also known to play important roles in hypercoagulation and thrombosis [46, 47]; and are changed in diabetes [19, 48, 49]. Either hypercoagulability [25–27, 50–54]; or reduced clot permeability; or decreased susceptibility of clots to undergo (fibrino)lysis, have previously been reported [21, 26, 28–31, 51, 54–58]. Increased glucose levels may also play an important role in fibrin fibre packaging, where glycation of fibrinogen may cause changes in structural packaging of fibrinogen into fibrin [32–34, 57].

Type 2 diabetes is therefore a complex disease associated with a multifaceted systemic inflammatory profile, and although the type 2 diabetes patients in our sample group were on a plethora of medication, in many cases, they were still poorly controlled. Despite the fact that many were also on anticoagulant treatment, their TEG coagulation profiles still varied greatly, suggesting that their coagulation processes were not correctly controlled.

In Tables 1 and 2, we showed all individual data of the controls and diabetes sample. This was done to stress that the individual type 2 diabetes sample results are greatly variable. Table 3 shows median and SD of all diabetes patients. Patient 72 to 90 only have R-values. A Tukey analysis was performed (it compares the means of every TEG parameter to the means of every other parameter) of all the TEG parameters of the controls to that of the diabetes sample. All comparative p-values were P > 0.9999. These results suggest that if diabetes data is compared using the total sample, without taking individual variability into account, we may loose valuable information regarding the individual patient. Therefore our findings support our plea to follow an individualized medicine approach in the treatment and disease tracking of diabetes type 2 patients. Our results also show that the coagulation profiles of our patients can be classified into four groups, as seen with TEG, and confirmed with SEM. Now, for the first time we show the possibility of a graded clotting classification, as the patients’ coagulation profiles vary from hypocoagulable to hypercoagulable. We acknowledge that the results indicated as the “hypocoagulable group” (shown as patient 72 to 90) are unusual.

We note that:

- Throughout the thrombelastographic literature, the only time one sees “flatline” TEG traces is after near fatal snakebite, liver transplantation, or life threatening coagulopathies of congenital or acquired conditions. However, none of these patients had these conditions.
- Also, the anticoagulant medication used by the diabetic group would be expected to affect platelets, not plasmatic coagulation kinetics.
- Even in patients with “flatline” TEG results, fibrin fibres could be formed when thrombin was added to the PPP, and their fibres did not look overly different from what we have seen in the “typical” diabetes population.
- We have never seen such traces in healthy individuals.

Possible reasons for such traces might be:

- The fibrinogen could have auto-polymerized in the citrated sample after thawing.
- Patients might have had carbon monoxide exposure; such an event removes fibrinogen from the sample, resulting in loss of TEG signal.
- Wrong laboratory procedures e.g. pipette usage when calcium was added.

Although we confirm that none of the above happened, we also do not have any physiological explanation for this phenomenon at present, except that a “false” “flatline” R-time is shown due to an inability of the TEG to initiate clotting. Previously it was reported that it is easier to dissolve clots that consist of fewer thick fibres than those that consist of many thin fibres, and this is consistent with experimental and clinical observations [59]. As mentioned, increased glucose levels may also play an important role in fibrin fibre packaging where glycation of fibrinogen may cause changes in structural packaging of fibrinogen into fibrin [32–34, 57]. Further studies regarding the coagulation process of type 2 diabetes is therefore suggested.

We conclude by suggesting that the only way to treat any type 2 diabetes patient, is to follow an *individualized patient tracking approach, instead of a generalized clinical approach.* Such an individualised approach using information from viscoelastic parameters may play a fundamental role in a precision-medicine approach, together with genome-based analysis and metabolomics data. We are currently not winning the race to reduce diabetes prevalence; to the contrary, our staggering type 2 diabetes and pre-diabetes statistics attest the fact that this disease is out of control [4, 5]. We therefore need to improve our understanding of inflammation and the role of abnormal clotting seen in type 2 diabetes patients. Clotting and coagulation in general, is an important marker of healthiness. However, if we use clotting potential as a marker of healthiness, we need to validate novel applications in clinical practice. Techniques like TEG, the global thrombosis test (GTT) and possibly also SEM may allow us to accomplish a truly individualized approach to diabetes patient care. Eventually, data from such techniques may ultimately be used in precision medicine.

## MATERIALS AND METHODS

Healthy individuals were screened and chosen to participate in the study if they did not have any chronic condition, did not smoke or if female, use any hormone replacement or contraception. Diabetic individuals were chosen arbitrarily from the diabetic clinic at the Steve Biko Academic Hospital, South Africa. The patients were diagnosed according to the SEMSDA guidelines (http://www.semdsa.org.za/files/Diabetes%20Guidelines%202009.pdf). These guidelines follow the American Diabetes Association (ADA) criteria to define type 2 diabetes. Citrated blood was collected for thromboelastography (TEG) and scanning electron microscopy studies (SEM). Ethical clearance was obtained from the Health Sciences Ethical Committee of the University of Pretoria.

Blood was collected in 4mL plastic citrate tubes and this collection and all handling of samples were performed under very strictly controlled aseptic conditions. A medical practitioner identified and recruited the patients, and collected the data regarding the medication usage. The same individual collected all the blood samples from the type 2 diabetes group, as well as the blood from the recruited healthy individuals. Citrated blood was left at room temperature for at least 30 minutes at no longer than 2 hours, followed by centrifuging and collecting of platelet poor plasma (PPP), which was immediately stored at −80 degrees C. On the day of the analysis, 340 μl of PPP was placed in a cup in the computer-controlled thrombelastograph® haemostasis system (Model 5000, Hemoscope, Niles, IL), together with 20 μl of 200 mM CaCl_2_, to initiate clotting. We ran control samples and diabetes samples on the same days, in order to minimize TEG variability. We also randomly (blinded) repeated analysis of samples to confirm results.

Data were collected until maximum elastic modulus (MG) is reached or 60 min had elapsed [60]. TEG traces show the following parameters: R: Reaction time, first measurable clot formation; K: Achievement of clot firmness; Angle: Kinetics of clot development; MA: Maximum clot strength; MRTG: Maximum rate of thrombus generation; TMRTG: Time to maximum rate of thrombus generation; TTG Final clot strength (for a detailed discussion of parameters also see Table 4). The StatsDirect program was used for statistical analysis.

**Table 4:** TEG parameters typically generated for platelet poor plasma [43, 61, 62]

THROMBOELASTIC PARAMETERS	
R value: reaction time measured in minutes	Time of latency from start of test to initial fibrin formation (amplitude of 2mm); i.e. initiation time
K: kinetics measured in minutes	Time taken to achieve a certain level of clot strength (amplitude of 20mm); i.e. amplification
Α (Alpha): Angle (slope between the traces represented by R and K) Angle is measured in degrees	The angle measures the speed at which fibrin build up and cross linking takes place, hence assesses the rate of clot formation; i.e. thrombin burst
MA: Maximal Amplitude measured in mm	Maximum strength/stiffness of clot. Reflects the ultimate strength of the fibrin clot, i.e. overall stability of the clot
Maximum rate of thrombus generation (MRTG) measured in Dyn.cm^−2^.s^−1^	The maximum velocity of clot growth observed or maximum rate of thrombus generation using G, where G is the elastic modulus strength of the thrombus in dynes per cm^−2^
Time to maximum rate of thrombus generation (TMRTG) measured in minutes	The time interval observed before the maximum speed of the clot growth
Total thrombus generation (TTG) measured in Dyn.cm^−2^	The clot strength: the amount of total resistance (to movement of the cup and pin) generated during clot formation. This is the total area under the velocity curve during clot growth, representing the amount of clot strength generated during clot growth

Extensive fibrin fibre networks for ultrastructural analysis were created using the same PPP stored for use in the TEG, by adding 20 μl of PPP to 10 μl thrombin (preparation as previously described [7]). Samples were viewed using a high-resolution crossbeam 540 Zeiss scanning electron microscope.
